# Peroxisome Proliferator-Activator Receptor **γ**: A Link between Macrophage CD36 and Inflammation in Malaria Infection

**DOI:** 10.1155/2012/640769

**Published:** 2012-01-11

**Authors:** Yi Ren

**Affiliations:** W. M. Keck Center for Collaborative Neuroscience, Rutgers, the State University of New Jersey, Nelson Labs D-251, 604 Allison Road, Piscataway, NJ 08854, USA

## Abstract

Severe malaria infection caused by *Plasmodium falciparum* is a global life-threatening disease and a leading cause of death worldwide. Intensive investigations have demonstrated that macrophages play crucial roles in control of inflammatory and immune responses and clearance of *Plasmodium-falciparum*-parasitized erythrocytes (PE). This paper focuses on how macrophage CD36 recognizes and internalizes PE and participates the inflammatory signaling in response to *Plasmodium falciparum*. In addition, recent advances in our current understanding of the biological actions of PPAR*γ* on CD36 and malaria clearance from the hosts are highlighted.

## 1. Introduction

Macrophages play a crucial role in the innate immunity [1] and are essential components of defense against the malaria infection. Macrophages can take up nonopsonic or opsonic *Plasmodium-falciparum*-parasitized erythrocytes (PE) by using CD36 and Fc receptors, respectively. Nonopsonic PE phagocytosis by macrophage CD36 plays a major role for PE clearance especially in the acute phase of primary malaria infection or nonimmune hosts [2, 3]. Therefore, upregulation of CD36 on macrophages could greatly trigger the capacity to clear parasites during acute malaria infection and thus is likely to be effective treatment for malaria infection. The intensive investigations demonstrate that peroxisome proliferator-activator receptor *γ* (PPAR*γ*) plays an important role in the immune response via inhibiting the expression of inflammatory cytokines and control macrophage alternative activation [4–6] and has potential as a novel anti-inflammatory target for many inflammatory diseases [7] including parasitic infection [8–10]. CD36 can be induced by PPAR*γ* ligands, and activation of PPAR*γ* enhances the clearance of PE and inhibits the proinflammatory response [6, 11]. Here, we highlight recent advances in our current understanding of the biological actions of PPAR*γ* on CD36 and malaria clearance from the hosts. 

## 2. CD36 and Malaria Clearance

Macrophages play a crucial role in innate immunity to malaria infection because they can phagocytose infected erythrocytes that limit the malaria density in the absence of cytophilic or opsonizing malaria-specific antibody [2]. Macrophage pattern-recognition receptors (PRRs), including Toll-like receptors (TLRs) and scavenger receptors such as CD36, are important components in the regulation of immune and inflammatory responses [12]. PRRs are a class of innate immune response-expressed proteins that recognize a wide range of molecules known as pathogen-associated molecular patterns (PAMPs) exposed on the pathogens or pathogen-infected cells but absent from healthy host cells and activate proinflammatory responses to infection. CD36, a member of the class B scavenger receptor family, has been identified as a PRR [12]. CD36 is an 88-kDa membrane glycoprotein that expressed on a wide range of cells such as platelets, monocytes/macrophages, endothelial cells, smooth muscle cells, and other types of cells. CD36 expressed on macrophages is involved in recognition and engulfment of endogenously derived ligands such as apoptotic and senescent cells [13–16], thrombospondin-1 [17, 18], nonopsonized bacteria [19], *β*-amyloid [20], and oxidized low-density lipoproteins (oxLDL) [21]. Because CD36 is expressed by broad range of cells and involved in uptake of many self- or non-self-particles, it contributes to a varied list of physiological and pathologic processes such as apoptotic cell clearance, angiogenesis, atherosclerosis, Alzheimer's disease and infectious diseases [22].

Macrophage CD36 is also involved in recognizing and internalizing nonopsonized *Plasmodium falciparum* parasitized erythrocytes (PE) and help control replication of blood-stage parasites, which is a critical component of host defense mechanisms against blood-stage parasites [3, 23–28]. African populations contain high frequency of mutations in CD36. These mutations that cause CD36 deficiency are associated with susceptibility to severe malaria and cerebral malaria [29]. Nonopsonized PE by antibodies or complements can be recognized and internalized by macrophages via CD36 because *Plasmodium falciparum *erythrocyte membrane protein 1 (PfEMP1) expressed on the surface of PE is the major parasite ligand for CD36 [2]. CD36 null mice show the importance of CD36 in PE phagocytosis [2]. Macrophages from CD36 null mice or rat lacking CD36 internalize significantly fewer PE compared to wild-type mice or control rats [2]. CD36-null mice experience more severe and fatal malaria when challenged with *Plasmodium chabaudi chabaudi *as compared with wild-type mice [30]. CD36-null mice also display defect in parasite clearance, earlier peak parasitemias, higher parasite densities, and higher mortality rates compared to wild-type mice [30]. These results suggest that selectively triggering of malaria clearance by modulating CD36 expression may contribute to control of acute blood-stage malaria infection *in vivo*.

Current data suggest that TLRs do not function directly as phagocytic receptors [31]. Erdman et al. applied selective, receptor-targeted strategies to show that macrophage pretreatment with TLR agonists markedly stimulates PE uptake via CD36, suggesting that CD36 and TLRs cooperate functionally to promote internalization of PE by macrophages [27]. The TLR2 activation-enhanced phagocytosis capacity for PE is unlikely via TLR-mediated transcription of scavenger receptors because surface CD36 levels do not increase upon TLR2 activation [27]. The role of TLR2 for the promotion of malaria clearance by macrophages may be beneficial in the control of acute blood stage parasite replication. Increasing understanding of the molecular mechanisms involved may lead to development of strategies for manipulating phagocytic clearance of *Plasmodium falciparum* for the treatment malaria infection.

## 3. CD36 and Inflammatory Responses

Macrophage CD36 plays a very important role in the physiological process including apoptotic cell clearance and pathogenesis, of many diseases such as atherosclerosis, Alzheimer's disease and* Plasmodium falciparum* malaria infection. In addition to clearance of altered self- or non-self-components such as apoptotic cells, oxLDL, *β*-amyloid, and pathogens, macrophage CD36 may be involved in inflammatory signaling cascade by interacting with other PRRs such as TLRs, which may further increase macrophage phagocytosis capacity and dispose invaded pathogens or trigger the chronic inflammation in these diseases. However, the interaction between CD36 and TLRs is complex and the consequences of the interaction are not fully understood. Macrophage CD36 recognition and internalization of apoptotic cells fails to stimulate proinflammatory response from ingesting phagocytes but triggers an anti-inflammatory response which is mediated by the release of interleukin (IL)-10, transforming growth factor (TGF)-*β* [32, 33] and inhibition of tumor necrosis factor-*α* (TNF-*α*), IL-12, IL-1*β*, and IL-8 [33]. It was also demonstrated that macrophages recognition and phagocytosis of late apoptotic cells does not trigger the release of IL-8 and TNF-*α*. By contrast, macrophages response to necrotic cells, including secondarily necrotic cells derived from uncleared apoptotic cells, are perceived as proinflammatory [34]. Macrophage CD36-dependent signaling is involved in the pro-inflammatory effects of internalizing *β*-amyloid, oxLDL, and bacteria [35–37]. CD36 has been shown to cooperate with TLR2 in innate sensing and induceing of inflammatory cytokines in response to TLR2 agonists such as intact bacteria and bacterial ligands [38–40]. By activation of src-family kinases, MAP kinases, transcription factor nuclear factor *κ*B (NF-*κ*B), macrophage CD36 recognition and internalization of oxLDL activates pro-inflammatory signals such as release of cytokines and production of reactive oxygen species (ROS) and inhibits macrophage migration, which results in the formation of foam cells and atherosclerotic plaque [12, 41]. For a comprehensive review on CD36-induced signaling pathways, see Moore and Freeman and Silverstein et al. [22, 41]. Microglia cell CD36 for *β*-amyloid uptake also boosts pro-inflammatory response and may contribute to the pathogenesis of Alzheimer's disease [12]. CD36 recognition of oxLDL and *β*-amyloid triggers assembly of a heterotrimeric complex composed of CD36, TLR4 and TLR6, leading to the induction of pro-inflammatory mediators implicated in the deleterious effects oxLDL and amyloid-*βin vivo *[42]. TLR2 also requires CD36 for inflammatory signaling [12]. CD36 is necessary for the component of ischemic brain injury attributable to the inflammatory response triggered by TLR2/1 activation [43]. The inflammatory response in brain induced by TLR2/1 activation, but not TLR2/6 or TLR4 activation, is suppressed in CD36-null mice. In contrast to brain inflammation, in systemic inflammation CD36 is involved in TLR2/6 activation, but not TLR2/1 activation [39, 40, 43]. This finding suggests that CD36 signaling in neuroinflammation differs from systemic inflammation; the mechanism for such differences is unclear [43].

Macrophage CD36 also contributes to the induction of innate immunity to malaria infection [30]. Macrophage CD36 interaction with malaria and malaria products, such as *Plasmodium falciparum *glycosylphosphatidylinositol (*pf*GPI), hemozoin and *Plasmodium falciparum *DNA, known as pathogen-associated molecular patterns (PAMPs), has been found to stimulate macrophage cytokine production via collaboration with TLR family [44–46].* pf*GPI is the primary parasite-derived bioactive molecule that activates macrophages and induces the release of inflammatory cytokines such as TNF-*α* and IL-1 from macrophages and, therefore, contributes to severe malarial pathogenesis and morbidity [11, 47]. *pf*GPI interacts with macrophage TLR2 by activation of JNK, P38, c-Jun and ERK1/2 in a CD36-dependent manner [30, 48]. Hemozoin, also known as malaria pigment, is insoluble crystal generated from hemoglobin proteolysis by *Plasmodium falciparum. *Natural hemozoin is coated with both proteins and plasmodial DNA and a potent activator of the inflammatory response via triggering TLR9 dependent on MyD88 [49, 50]. However, some studies show that the inflammatory effect of hemozoin is dependent on the cells, duration of incubation time, and the method for hemozoin preparation [51]. A recent study demonstrated that a PAMP, AT-rich DNA in the genome of *Plasmodium falciparum*, couples to stimulator of interferon genes (STING) and TANK-binding kinase (TBK) to induce interferon regulatory factor 3–7 (IRF3-IRF7) dependent on type I IFN production in a TLR9-independent manner. Mice lacking IRF3, IRF7, the kinase TBK1, or the type I IFN receptor were resistant to lethal cerebral malaria [46]. This study provides evidence of a unique DNA sensing pathway that may contribute to immunopathology in plasmodial infections [52].

The role of CD36 in inflammatory signals in response to malaria infection remains controversial. Several lines of evidence suggest that selective ligation of CD36 does not lead to proinflammatory cytokine production by macrophages [3, 28]. Erdman et al. applied selective, receptor-targeted strategies to assess whether CD36 and TLRs can cooperatively mediate immune response to PE. They demonstrate that targeted activation and internalization of CD36 fail to stimulate proinflammatory cytokine production [27]. CD36-mediated intact PE internalization is also noninflammatory even in the presence of TLR agonists [27]. These data suggest that it is possible that CD36 is not directly involved in regulation of pro-inflammatory response to* Plasmodium falciparum* but rather presents or concentrates ligands for recognition by other signaling receptors such as TLRs [30], similar to the role of CD14 in presenting or concentrating LPS signal to TLR4 [53]. Taken together, the contradictory data about CD36 on inflammatory response to parasite and parasite produce indicate that further investigation is needed for the role of CD36 in parasitic infection.

## 4. PPAR*γ* Ligand

PPARs are nuclear receptors which are ligand-activated transcription factors. To date, three different PPAR subtypes have been identified PPAR*α*, PPAR*β*/*δ*, and PPAR*γ*. PPAR*γ* was first identified for its role in lipid and glucose metabolism. PPAR*γ* is critical in a variety of biological processes. Natural and synthetic ligands bind to PPAR*γ*, resulting in conformational change and activation of PPAR*γ*.

**Natural Ligands**

Prostaglandin 15-deoxy-Δ12,14-prostaglandin J_2_ (15d-PGJ_2_) was the first discovered endogenous ligand for PPAR*γ* [54, 55]. 15d-PGJ_2_ is a potent activator for PPAR*γ* and responsible for many of anti-inflammatory actions [56]. The variety lipophilic ligands can bind and activate PPAR*γ*. The essential fatty acids, arachidonic acid, gamolenic acid, docosahexanoic acid, eicosapentaenoic acid, and components of oxLDL, such as 9,13-hydroxyoctadecadienoic acid (HODE) and 8,15-hydroxyeicosatetraenoic *acid* (HETE) are also potent endogenous activators of PPAR*γ* [57]. These PPAR*γ* ligands can be regulated by lipoxygenase and cytokines. For example, 13-HODE and 15-HETE can be generated from linoleic and arachidonic acids, respectively, by a 12/15-lipoxygenase that is upregulated by interleukin-4 [5, 58]. *Plasmodium falciparum* may itself activate PPAR*γ*. *Plasmodium falciparum* produces hemozoin, which induces the release of lipoxin A_4_ (LXA_4_), 5,15-diHETE, and 15-HETE that can activate PPAR [59].

**Synthetic PPAR*γ* Ligands**

In addition to natural ligands, many synthetic ligands have been identified. The anti-diabetic drugs thiazolidinediones (TZDs) including troglitazone, rosiglitazone, pioglitazone, and ciglitazone, used for the treatment of type 2 diabetes, are synthetic PPAR*γ* agonists [7, 60]. They regulate the expression of genes that are involved in lipid metabolism and insulin action by activation of PPAR*γ*. Furthermore, TZDs have beneficial anti-inflammatory properties that are widely used for treatment of patients with inflammatory diseases [7].

## 5. PPAR*γ* and CD36

Macrophage and macrophage CD36 play a critical role in clearing PE and protecting host from high density of parasites during acute blood-stage malaria infection [28, 30, 61]. Therefore, upregulation of CD36 expression on macrophages may trigger parasite clearance and enhance survival of the host. CD36 can be upregulated by different molecules. Treatment macrophages with oxLDL and inflammatory cytokines such as granulocyte macrophage colony-stimulating factor (GM-CSF), macrophage colony-stimulating factor (M-CSF), upregulate CD36 expression [62]. The mechanism of induction of CD36 by oxLDL and these cytokines is due to the ability to activate PPAR*γ* [63]. In addition, both IL-13 and IL-4, which induce an alternative activation of macrophages [64], induce expression of CD36 by generation of natural ligands of PPAR*γ* [5, 65]. The functional consequences of CD36 expression and PPAR*γ* activation induced by IL-13 are to enhance phagocytosis of PE [65]. The role of PPAR*γ* in CD36 expression is further confirmed by using macrophages lacking expression of PPAR*γ*. By using macrophages differentiated from PPAR*γ*-deficient embryonic stem cells, PPAR*γ* is not essential for macrophage differentiation, but it is required for basal expression of CD36 [66] and necessary for the regulation of CD36 in response to PPAR*γ* ligands in PPAR*γ*-deficient stem cell-differentiated macrophages [67]. IL-13 up-regulates macrophage CD36 expression, and it is ineffective on CD36 induction on PPAR*γ*-deficient macrophages compared to wild-type cells [65]. It is particularly interesting because IL-13 not only induces macrophage alternative activation [64] which could help control parasite burden while limiting associated inflammation and thus reducing host pathology, but also enhances CD36-dependent PE phagocytosis without TLR2 involvement [65].

Incubation of macrophages with PPAR-RXR agonists, including 15d-PGJ2, 9-*cis*-retinoic acid (9-*cis*-RA), and TZD increase CD36 expression on macrophages which associates with increased capacity for phagocytosis of PE [28]. This increase in phagocytosis of PE is accompanied by a decrease in parasite-induced TNF-*α* production. PPAR*γ* agonist rosiglitazone enhances phagocytic clearance of PE and inhibits inflammatory responses to infection via inhibition of *pf*GPI-induced activation of the MAPK and NF-*κ*B signaling pathways [8]. Furthermore, rosiglitazone reduces the parasitemia in a CD36-dependent manner in the *Plasmodium chabaudi chabaudi *hyperparasitemia model and improves the survival rate even when treatment is initiated as late as day 5 after infection [8]. These results indicate that specific upregulation of CD36 by these compounds may represent a novel means for modulating host clearance of PE and proinflammatory responses to *Plasmodium falciparum *[28]. Despite studies about PPAR*γ* agonists that enhance CD36 expression and uptake PE, this treatment raises concerns that treating individual with PPAR*γ* agonists could also enhance CD36 expression on endothelial cells and thus triggers the adherence of PE to endothelial cells in various blood vessels. Therefore, specific upregulation of CD36 expression on macrophages may represent a novel therapeutic strategy to treat malaria infections.
